# Supplementary material for: Associative detachment in anion-atom reactions involving a dipole-bound electron
Source: Nat Commun. 2022 Feb 10;13:818. doi: 10.1038/s41467-022-28382-w (PMC8831523; doi:10.1038/s41467-022-28382-w)
Supplement: Supplementary file 1 — Supplementary Information [file 41467_2022_28382_MOESM1_ESM.pdf]

Supplementary Information

Associative detachment in anion-atom reactions involving a dipole-bound electron

Saba Zia Hassan[1], Jonas Tauch[1], Milaim Kas[2,3], Markus Nötzold[4], Henry López Carrera[1†], Eric S. Endres[1,4], Roland Wester[4], and Matthias Weidemüller[1*]

[1] *Physikalisches Institut, Ruprecht-Karls-Universität Heidelberg, 69120 Heidelberg, Germany*
[2] *Département de Chimie, Faculté des Sciences, Université Libre de Bruxelles (ULB), 1050 Brusseles, Belgium*
[3] *Deutsches Elektronen-Synchrotron (DESY), 22607 Hamburg, Germany*
[4] *Institut für Ionenphysik und Angewandte Physik, Universität Innsbruck, 6020 Innsbruck, Austria*
[*] *Corresponding author: weidemueller@uni-heidelberg.de*
[†] *Current address: Universidad de Las Fuerzas Armadas ESPE, 171103 Sangolquí, Ecuador*

December 15, 2021

Supplementary Note 1

Ab-initio potential energy surfaces

Multi-configuration approaches have been used in order to obtain the excited potential energy surfaces that correlate to the Rb($P_{3/2}$)+OH^- entrance channel. We have accounted for the spin-orbit coupling using the state interacting method as implemented in the MOLPRO [1] program using the spin-orbit operator from the MDF effective core potential. A total of 6 interacting anionic states and 2 neutral states have been taken into account. A set of state averaged orbitals was obtained by performing multi-configuration calculation on selected configurations [2]. These orbitals were then used to perform configuration interaction using the internally contracted scheme (ic-MRCI) implemented in the MOLPRO program, including the Davidson correction (labelled +Q) [3].
The ic-MRCI+Q has been applied to a set of manually selected configurations, in the C_s point group. The selected configurations have been obtained from a separate SA-CASSCF calculation on the four A' and two A'' low lying electronic states of the $RbOH^-$ molecular complex, taken in its optimized geometry (linear geometry). The active space covers $9a'$ and $3a''$ molecular orbitals (MO).
Singly excited configurations were found to be dominant contributors in the CI expansion. This is expected from electronic configurations consideration. The ground-state electronic configuration is $1a'^2 2a'^2 3a'^2 1a''^2 4a'^2 5a'^2 6a'^2 7a'^2 2a''^2 8a'^1$, where the $1a'$, $2a'$ and $3a'$ MOs correspond to the $1s_O$, $4s_{Rb}$ and $2s_O$ atomic orbital (AO), respectively, the $4a'$, $1a''$, $5a'$ and $6a'$, $7a'$, $2a''$ MOs are mainly formed by the $4p_{Rb}$ and $2p_O$ AOs, respectively, and the $8a'$ MO is mainly formed by the $5s_{Rb}$ AO and corresponds to the highest occupied MO (HOMO). In further discussion we will omit the $1a'^2 2a'^2 3a'^2 1a''^2 4a'^2 5a'^2 6a'^2$ inner shell, which will be labelled with brackets [], and only focus on the valence MOs. The first unoccupied MOs, $9a'$, $10a'$ and $3a''$ are mainly formed by the $5p_{Rb}$ AOs, the ground-state electronic configuration becomes $[]7a'^2 2a''^2 8a'^1 9a'^0 10a'^0 3a''^0$. The first four A' and two A'' low lying excited states of the $RbOH^-$ molecular complex correlate to the following channels: $Rb^-(^1S)+OH(^2\Pi)$, $Rb(^2S)+OH^-(^1\Sigma^+)$ and $Rb(^2P)+OH^-(^1\Sigma^+)$. Their main electronic configuration correspond to single excitation relative to the ground-state configuration within the valence $7a' 2a'' 8a' 9a' 10a' 3a''$ MOs. The different electronic states along with their main electronic configurations are given in Supplementary Table 1. The dissociation channels at which they diabatically correlate are also depicted. It should be noted that these electronic excited states correspond to shape resonances of the RbOH+e^- collisional system.

**Electronic states**	**Main electronic config.**	**Dissociation Height**
$X\,^2A'$	$[]7a'^2 2a''^2 8a'^1$	$Rb(^2S)+OH^-(^1\Sigma^+)$
$2\,^2A' \oplus 1\,^2A''$	$[]7a'^2 2a''^2 8a'^0 9a'^1 \oplus []7a'^2 2a''^2 8a'^0 3a''^1$	$Rb(^2P)+OH^-(^1\Sigma^+)$
$3\,^2A'$	$[]7a'^2 2a''^2 8a'^0 10a'^1$	
$4\,^2A' \oplus 2\,^2A''$	$[]7a'^1 2a''^2 8a'^2 \oplus []7a'^2 2a''^1 8a'^2$	$Rb^-(^1S)+OH(^2\Pi)$

Supplementary Table 1: The main electronic configurations and dissociation channels of different electronic states are presented.

A particularly interesting feature of the Rb-OH^- collisional complex is the drastic change in the binding nature of the excess electron along the reaction path. The excess electron is first localized on the hydroxyl anion, which is a typical closed shell valence-bound anion, characterized by a compact density, large detachment energies (≈ 1.8 eV) and important electron correlation effects. When the reaction proceed, the Rb atom disturbs the electron cloud, the excess electron becomes more diffuse and less strongly bound

where it is primary bound via dipole-charge interaction.
One of the usual difficulties one faces while using standard bound-state quantum chemistry approaches to calculated metastable states embedded in a continuum is that the wave function usually undergoes variational collapses to a neutral + free electron state where the excess electron occupies a very diffuse orbital. Various stabilization schemes can be used to tackle this problem. Here we found out that constraining the reference MCSCF wave function to single excited configurations avoids the convergence problems and allows us to obtain the resonance states even when using diffuse basis set. The potential energy surfaces (PESs) shown in the main text have been obtained with this manually selected MCSCF/ic-MRCI approach using the MDF ECP for Rb along with its *spdfg* companion basis set [4] supplemented by a set of $5s4p3d2fg$ even tempered diffuse functions and the AVQZ basis set for the O and H atom [5].
Using group theory consideration the four $^1A'$ + two $^1A''$ electronic states result in six interacting $E_{1/2}$ spin-orbit (SO) states. The results are shown in Supplementary Figure 1 for $\theta = 80°$. The two insets show the behaviour near the potential well and at dissociation, lower and upper left panels, respectively. The PES of the neutral RbOH ground-state is shown in black line. Our calculation correctly reproduces the SO splitting of the $^2P_{3/2}$ and $^2P_{1/2}$ state of Rb, with a calculated value of 26.5 meV which is to be compared to experimental value 29.3 meV [6].

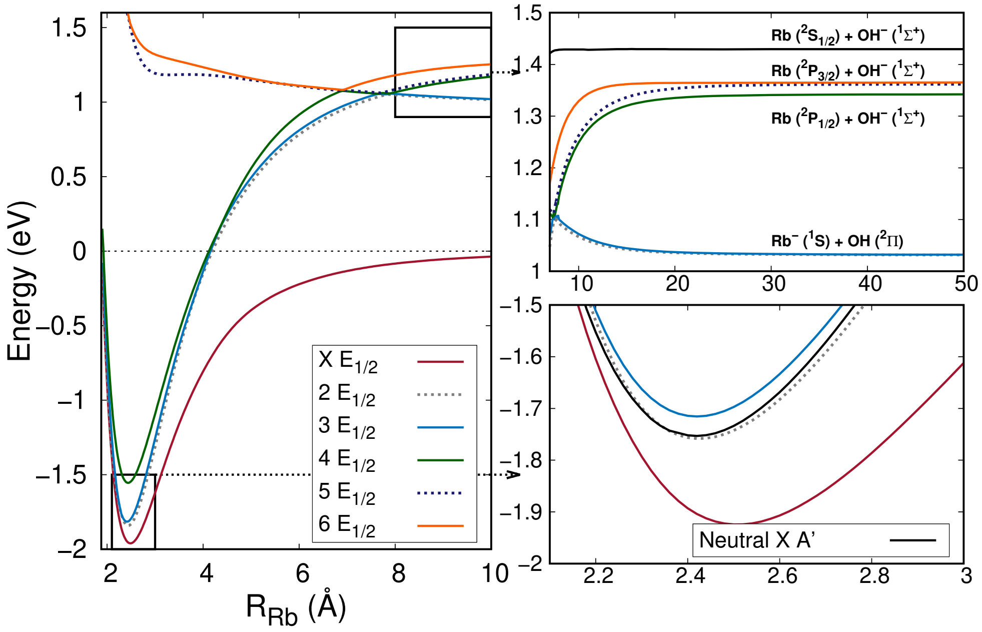

Supplementary Figure 1: Left panel: PESs of the low-lying excited states of the Rb-OH$^-$ molecular complex for $\theta = 80°$, including SO couplings. This leads to 6 interacting $E_{1/2}$ states. The red curve and other solid colored lines correspond to the adiabatic potential of the ground- and excited-states of the Rb-OH$^-$ collision complex, respectively. The dashed lines correspond to the A" states in the non-relativistic picture. The upper right panel show the PESs of the excited states at dissociation where the relevant channels are depicted. The bottom right panel shows the PESs of the 3 lowest anionic states (red, dashed gray and blue curve) around the potential well alongside the neutral potential (black curve).

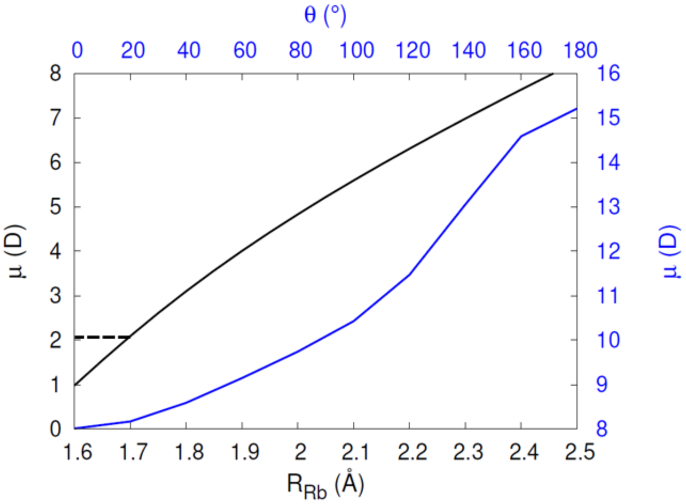

Supplementary Figure 2: Hartree-Fock calculation of the dipole moment of RbOH. The black curve shows the dipole moment μ as a function of internuclear distance R_{Rb} for $\theta = 0°$. The blue curve shows the dipole moment as a function of collisional angle θ for an optimized internuclear distance. The critical value of the dipole moment ensuring a stable intermediate dipolar complex is indicated by the dashed line.

Supplementary Note 2

Features of the potential energy surface

2.1 Potential well region

Our calculations show that the excited states of the $RbOH^-$ complex, taken in its equilibrium geometry ($\theta = 0°$) are auto-detaching states, in other words, only the ground state has a positive vertical detachment energy (VDE). However, for larger values of θ dipole moment of the RbOH core increases, leading to a stabilization of the anion. This can be seen in Supplementary Figure 2, where the the Hartree-Fock dipole moment of the RbOH core is depicted as a function of R_{Rb} for $\theta = 0°$ (black) and as a function of θ at optimized R_{Rb} distance (blue). As a consequence, the VDE increases for increasing θ. This trend can be seen in Supplementary Figure 3 where the crossing point between the anionic first excited-state $2E_{1/2}$ PES and the neutral PES is marked by a dot. In particular, for $\theta > 153°$ the crossing with the neutral PES occurs above the $Rb(^2P_{3/2})$+$OH^-(^1\Sigma^+)$ entrance channel energy. The later is taken as $E_c + T(\mu, J)$, where E_c and $T(\mu, J)$ are the collision energy and internal energy (vibration and rotation) of OH^- at $T = 355$K, respectively. This characterizes an accessible angular space where the auto-detachment from the $2E_{1/2}$ is avoided.

2.2 Long and intermediate range

Although it is difficult to see in the Supplementary Figure 1, the $6E_{1/2}$ state is repulsive at long range and attractive at intermediate distance, leading to a potential barrier around 14 Å. The barrier can be seen in Supplementary Figure 4 where the PESs for the $6E_{1/2}$ state for various values of θ have been plotted. The barrier height is almost independent of θ and is about 4.5×10^{-3} eV high. This repulsive state can be related to the classical repulsive potential of a charge-quadrupole interaction [7]. We suspect the presence of a higher lying state, with a repulsive potential at intermediate and short range, that interacts with the $6E_{1/2}$ state leading to an avoided crossing. We found this state

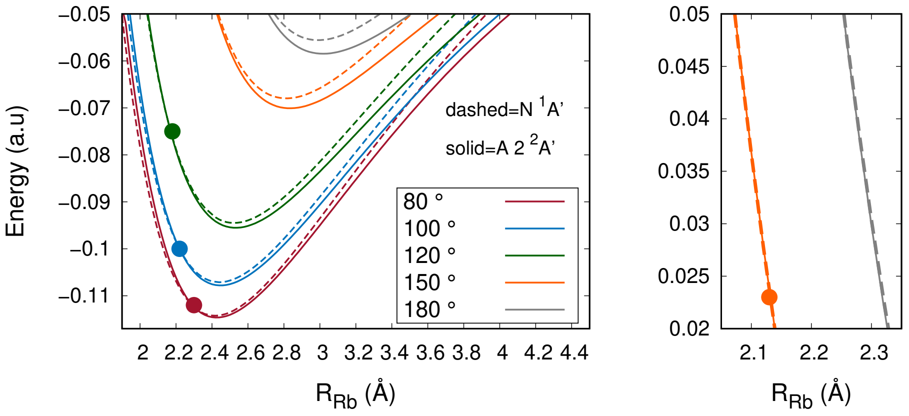

Supplementary Figure 3: PESs for the first excited state of the anion $RbOH^-$ ($2\,^2A'$ or $2\,E_{1/2}$, solid lines) and the ground state of the neutral RbOH molecular complex (dashed lines), for various collision angle θ. Note that the colors corresponds to various values of θ. The right panel shows a zoom-in of the PESs in the repulsive region. The zero energy is taken as the energy of the ground state entrance channel $Rb(^2S)+OH^-$.

to be mainly described by doubly excited electronic configurations, hence it corresponds to a Feshbach resonance of the RbOH+e^- collisional complex. Our approach based on manually selected singly excited configurations is not adequate to describe this additional state. Unfortunately, due to the convergence problems pointed out above, adding even the minimum number of doubly excited configurations needed to describe this additional state results in variational collapses of the wave function. Therefore, specific methods would be needed.

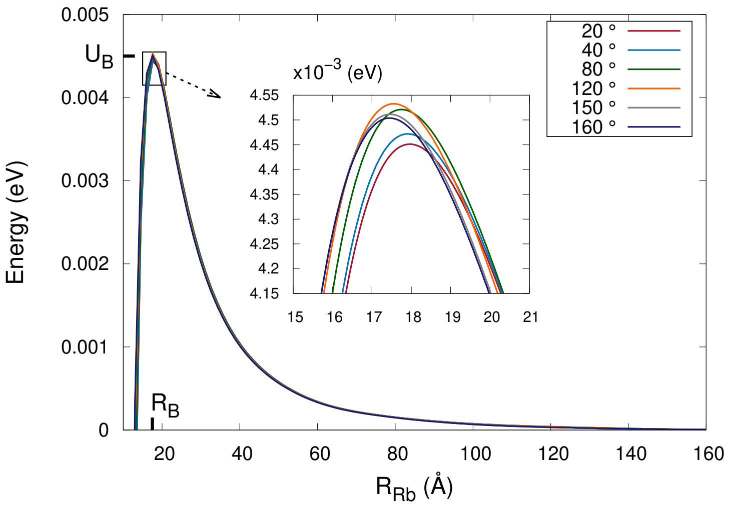

Supplementary Figure 4: Potential barrier along the $6E_{1/2}$ state for various values of θ. The distance R_B is the intermolecular distance R_{Rb} at the potential's maximum value U_B.

Supplementary Note 3

Determination of Langevin reaction rate

In the event of an atom-ion collision, the upper limit to collisional rate constants is given by the classical-mechanics based, Langevin capture model [8]. The long range interaction potential V(r) is dominated by the interaction between charge-induced dipole of the atom with the ion's charge such that:

$$V_{\text{int}}(r) = -\frac{C_4}{r^4} \tag{1}$$

where r is the interparticle separation and $C_4 = \frac{\alpha e^2}{(4\pi\epsilon_0)^2}$ [9]. Here α is the scalar polarizability of the neutral, e is the electron charge and ϵ_0 is the permittivity of free space.
By including the centrifugal potential into the effective potential of the collision complex, the Langevin reaction rate constant k_{L} can be derived as :

$$k_{\text{L}} = 2\pi\sqrt{\frac{C_4}{\mu}} = \frac{e}{2\epsilon_0}\sqrt{\frac{\alpha}{\mu}} \tag{2}$$

where all parameters are in S.I. units.
The scalar polarizabilities and the calculated reaction rate constants for excited and ground-state Rb are summarized as follows:

	α (a.u.) [10]	k_{L} (10^{-9} cm^3s^{-1})
Rb (^{2}P)	870	7.2
Rb (^{2}S)	318.6	4.3

Note: Conversion factor used for α (from atomic units to S.I. units) [10], 1 a.u. = 1.648773×10^{-41} $\text{C}\,\text{m}^2\,\text{V}^{-1}$.
The modified Langevin model that has been used to calculate the loss rate from the ground and excited state channel averages over the range of angles of approach for which the reaction is exoergic. There are some implicit assumptions in this model, which require some additional discussion

1. The present model assumes that no angle of approach is preferred. Steering effects, i.e. reorientation of the OH^- molecule due to intermolecular forces, start to play a role at low temperatures and would lead to an increase in the likelihood of a "head-on" collision of Rb-O-H, thus increasing the AED rate towards the Langevin rate. Although we do not expect these effects to be dominant in the temperature regime of our experiment, they might partially explain the underestimation of the measured rate coefficient by the *ab initio* calculation.

2. The population of rotational levels in OH^- may not follow a thermal distribution due to collisional cooling with the ultracold buffer gas. However, it has been shown that a lower rotational temperature would actually decrease the measured AED rate [11].

3. As suggested in [12], the presence of vibrational excited OH^- anions will strongly increase the AED loss. Using the dipole moment of OH^-, we find that the lifetime of $v = 1$ state, before radiative decay is on the order of couple of milliseconds, which in comparison to the complete timescale of the experiment ($\sim$ 10 seconds), makes the fraction of ions in the excited vibrational states negligible, as mentioned in the main text.

Supplementary Note 4

Excited-state loss channels

The interaction of hydroxyl anion with excited Rb (^{2}P) opens the following loss channels:

- **Associative electronic detachment (AED):**

$$\mathrm{Rb(^2P) + OH^- \rightarrow RbOH + e^-}$$

- **Electronic to kinetic energy transfer:**

$$\mathrm{Rb(^2P) + OH^- \rightarrow Rb + OH^- + E}_{kin}$$

- **Charge-exchange reaction:**

$$\mathrm{Rb(^2P) + OH^- \rightarrow Rb^- + OH}$$

4.1 Electronic to kinetic energy transfer

In order to estimate the probability to exit through the electronic to kinetic energy transfer $\mathrm{Rb(^2P_{3/2}){+}OH^- \rightarrow Rb(^2S){+}OH^-}$ channel, we have calculated the non-adiabatic coupling matrix elements (NACME) between the $\mathrm{XE_{1/2}}$ and $\mathrm{2E_{1/2}}$ states, which link to entrance and exit channels. This has been done using the finite difference approach, implemented in MOLPRO. The results can be seen in Supplementary Figure 5. The coupling exhibit

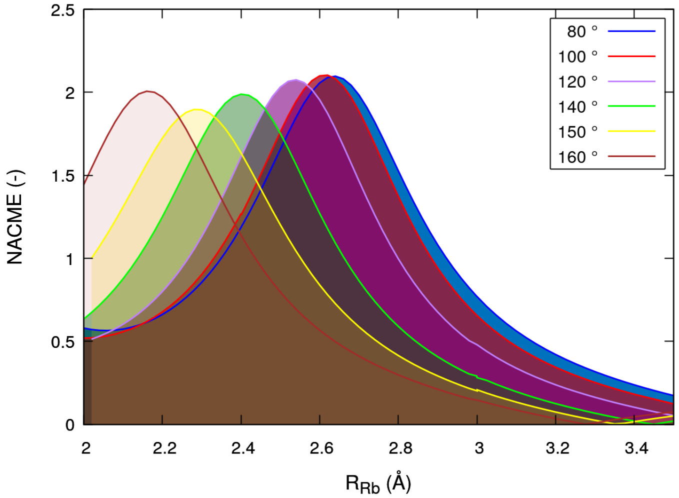

Supplementary Figure 5: Non-adiabatic coupling strength between the $\mathrm{XE_{1/2}}$ and $\mathrm{2E_{1/2}}$ states of the Rb-OH$^-$ molecular complex for various θ.

the typical bell-shape behaviour of non-crossing states with constant energy spacing. This coupling case can be described by the Rosen-Zener-Demkov model [13, 14] for which the coupling potential is modeled by a hyperbolic secant:

$$V_{12}(\mathrm{R_{Rb}}) = v_0 sech(\frac{\mathrm{R_{Rb}} - \mathrm{R_0}}{\beta}) \quad (3)$$

and the probability transition between adiabatic states (in atomic units) is given by

$$P_{12} = sin^2(\pi v_0 \beta) sech^2(\frac{\pi \varDelta E \beta}{2\sqrt{2E_k/\mu}}) \quad (4)$$

where $\varDelta E$ is the energy difference between both non-crossing adiabatic states, E_k is the kinetic energy. The parameters β, R_{Rb0} and v_0 were extracted by fitting our NACME calculation. The fitted values are $v_0 = 1.95$, $R_{Rb0} = 4.79$ and $\beta = 0.63$. With $\varDelta E \approx 7 \times 10^{-3}, E_k \approx 0.056$ we found the non-adiabatic probability transition $P_{12} \approx 0.15\%$ for the relevant collision energies. This small probability is mainly due to the large reduced mass of the system and the larger energy gap between adiabatic states in comparison with the avoided crossing case. This justifies the neglect of the electronic to kinetic energy transfer channel in the dynamics.

4.2 Charge transfer channel

The probability for an adiabatic passage at the various avoided crossings (at $R_{Rb} \approx 8$ Å, 7 Å and 4 Å in Supplementary Figure 1, left panel) is estimated using the Landau-Zener formula:

$$P_{12} = exp(\frac{\pi(\varDelta E)^2}{2\varDelta F\sqrt{(2E_k/\mu)}}) \quad (5)$$

where $\varDelta E$ and E_k are the energy gap and kinetic energy at the avoided crossing point respectively, μ is the reduced mass and $\varDelta F$ is the difference in the slope between the two diabatic curves. We found the diabatic transition probability P_{12} to be close to one for the relevant collision energies. This is primarily due to the small energy gap between the adiabatic states. Typical value of P_{12} are found to be around 0.95.

The probability to exit via the charge transfer channel Rb^-+OH is given by $P_{CT} = 1 - P_{NR}$, where P_{CT} is obtained by summing the probabilities associated to different paths that lead to the non-reactive channel. Owing to the highly diabatic nature of the PES (*i.e* small Landau-Zener adiabatic transition probability, see above), P_{CT} is very small, around 1.5% for the relevant collision energies. Since the loss from the electronic to kinetic energy transfer channel is also found to be small, the AED reaction is predicted to be the dominant loss channel.

Supplementary References

[1] Berning, A., Schweizer, M., Werner, H.-J., Knowles, P. J. & Palmieri, P. Spin-orbit matrix elements for internally contracted multireference configuration interaction wavefunctions. *Mol. Phys.* **98**, 1823–1833 (2000).

[2] Knowles, P. J. & Werner, H.-J. Internally contracted multiconfiguration-reference configuration interaction calculations for excited states. *Theor. Chim. Acta* **84**, 95–103 (1992).

[3] Langhoff, S. R. & Davidson, E. R. Configuration interaction calculations on the nitrogen molecule. *Int. J. Quantum Chem.* **8**, 61–72 (1974).

[4] Lim, I. S., Schwerdtfeger, P., Metz, B. & Stoll, H. All-electron and relativistic pseudopotential studies for the group 1 element polarizabilities from K to element 119. *J. Chem. Phys.* **122**, 104103 (2005).

[5] Dunning, T. H. Gaussian basis sets for use in correlated molecular calculations. I. The atoms boron through neon and hydrogen. *J. Chem. Phys.* **90**, 1007 (1989).

[6] Sansonetti, J. E. Wavelengths, Transition Probabilities, and Energy Levels for the Spectra of Rubidium (Rb I through Rb XXXVII). *J. Phys. Chem. Ref. Data* **35**, 301 (2006).

[7] Mies, F. H. Molecular theory of atomic collisions: Fine-structure transitions. *Phys. Rev. A* **7**, 942–957 (1973).

[8] Langevin, P. A fundamental formula of kinetic theory. *Ann. Chim. Phys.* **5**, 245 (1905).

[9] Vogt, E. & Wannier, G. H. Scattering of ions by polarization forces. *Phys. Rev.* **95**, 1190–1198 (1954).

[10] Mitroy, J., Safronova, M. S. & Clark, C. W. Theory and applications of atomic and ionic polarizabilities. *J. Phys. B: At. Mol. Opt. Phys.* **43**, 202001 (2010).

[11] Kas, M., Loreau, J., Liévin, J. & Vaeck, N. Ab initio study of reactive collisions between Rb(^{2}S) or Rb(^{2}P) and $OH^-(1\Sigma^+)$. *J. Chem. Phys.* **144**, 204306 (2016).

[12] Byrd, J. N., Michels, H. H., Montgomery, J. A. & Côté, R. Associative detachment of rubidium hydroxide. *Phys. Rev. A - At. Mol. Opt. Phys.* **88**, 032710 (2013).

[13] Nikitin, E. E. Nonadiabatic transitions what we learned from old masters and how much we owe them. *Annu. Rev. Phys. Chem. 1999.* **50**, 1 (1999).

[14] Desouter-Lecomte, M. & Lorquet, J. C. Nonadiabatic interactions in unimolecular decay. II. Simplified formalism. *J. Chem. Phys.* **66**, 4006–4017 (1977).
